# Investigation of an efficient multi-modal convolutional neural network for multiple sclerosis lesion detection

**DOI:** 10.1038/s41598-023-48578-4

**Published:** 2023-11-30

**Authors:** Florian Raab, Wilhelm Malloni, Simon Wein, Mark W. Greenlee, Elmar W. Lang

**Affiliations:** 1 Computational Intelligence and Machine Learning Group, University of Regensburg, 93051 Regensburg, Germany; 2 Experimental Psychology, University of Regensburg, Regensburg, 93051 Germany

**Keywords:** Image processing, Neurological disorders, Information technology

## Abstract

In this study, an automated 2D machine learning approach for fast and precise segmentation of MS lesions from multi-modal magnetic resonance images (mmMRI) is presented. The method is based on an U-Net like convolutional neural network (CNN) for automated 2D slice-based-segmentation of brain MRI volumes. The individual modalities are encoded in separate downsampling branches without weight sharing, to leverage the specific features. Skip connections input feature maps to multi-scale feature fusion (MSFF) blocks at every decoder stage of the network. Those are followed by multi-scale feature upsampling (MSFU) blocks which use the information about lesion shape and location. The CNN is evaluated on two publicly available datasets: The ISBI 2015 longitudinal MS lesion segmentation challenge dataset containing 19 subjects and the MICCAI 2016 MSSEG challenge dataset containing 15 subjects from various scanners. The proposed multi-input 2D architecture is among the top performing approaches in the ISBI challenge, to which open-access papers are available, is able to outperform state-of-the-art 3D approaches without additional post-processing, can be adapted to other scanners quickly, is robust against scanner variability and can be deployed for inference even on a standard laptop without a dedicated GPU.

## Introduction

Multiple sclerosis (MS) is the most frequently occurring immune-mediated inflammatory disease of the central nervous system in young adults. Magnetic resonance imaging (MRI) is a very important tool for diagnosis, treatment and follow up of this disease. For this reason, the lesions have to be exactly delineated^1^. This can be done manually by expert raters, but has a lot of practical downsides^2^. The other possibility is to use automated segmentation methods like the Lesion Segmentation Toolbox (LST) from SPM. However, the latter can only handle FLAIR + T1weighted images as input^3^. An improvement of automatic lesion segmentation can be achieved by incorporating deep learning methods that can learn new features from labeled training data and therefore can be tailored to the specific needs of a physician (e.g. in terms of available modalities and scanner variability). With respect to MS lesions, it is especially important to monitor all focal lesions visible on MRI sequences, even very small ones, as this is essential for disease staging, prognosis, and evaluating treatment efficacy. Deep learning methods, especially convolutional neural networks (CNNs), fully convolutional networks (FCN)^4^, generative adversarial networks (GAN)^5,6^ and encoder- decoder- based designs (autoencoders—AE)^7^ have come a long way in the last decade and were able to demonstrate outstanding performance in biomedical image analysis, where they provide state-of-the-art results for several problems^8–13^.

All these deep learning methods for image analysis can basically be subdivided into two different main groups: image-based and patch-based methods. The latter are frequently used in biomedical image analysis, due to the fact that most of the time, the available datasets are very small. Hence, the amount of training samples is increased considerably by extracting small patches of the original image, which serve as the input for a CNN. However, due to the small size of the patches, they neglect the global structure information and moreover, there is an increase in training and inference time^14^. Image based approaches process the whole image, exploiting the global structure information and can be implemented in a 3D- or 2D-based way. For the first, a CNN with 3D kernels is used, which is fed with the original 3D volume. With such kernels, the training process usually has to fit a very large number of parameters on the small dataset. This is prone to overfitting and the computational cost is also very high^15^. These problems can be reduced by a straight 2D implementation. There, the 3D volume is sliced into its corresponding 2D slices which are then processed individually by standard 2D convolution. The segmentations can then be reassembled into a 3D volume. This approach neglects contextual information in the plane orthogonal to the specific slice. However, this has less of a negative impact if this process is carried out for the axial, coronal and sagittal views and thus the final segmentation is generated from a combination of all three planes. The main improvement is the larger number of training samples (every slice of a volume instead of just one volume), which reduces the chance of overfitting. Also, the computational cost is drastically decreased compared to 3D approaches^14^.

For a good overview of the most promising approaches to segment MS Lesions, the best challenge submissions to the ISBI 2015 longitudinal MS Lesion Segmentation Challenge^16^ with published papers were taken into consideration. The today’s best performing approach is the *2.5 D Tiramisu* CNN^17^, which is basically a U-Net^8^ that has dense blocks and takes a stack of three adjacent 2D slices as input, to provide at least some contextual information in the third dimension. The second one on the leader board is the *ALL-NET*^18^, which is a cascaded CNN and consists of three parts. A 3D U-Net and two so-called Anatomical Convolutional Modules. On third and fourth place, there are two similar versions of the *nnU-Net*^19,20^, which is a combination of 2D and 3D U-Nets. *DeepLesionBrain*^21^ is on fifth place and is a 3D patch-based U-Net that is trained in two cascading steps. On rank seven, there is *IMAGINE*^22^, a 3D patch-based DenseNet^23^ with a U-Net like structure in the contracting-expanding stages, trained with an asymmetric similarity loss function based on the Tversky index. The eighth ranking approach is the *Self-adaptive network*^24^. This is a 3D patch-based U-Net implementation with sequence dropout. On rank 9 is a 2D slice-based multi-branch U-Net-like architecture with three parallel ResNets in the downsampling branches^25^. The last considered approach, the *Attention-Based CNN*^26^, lands on rank 10 and is a patch-based 3D ResNet with spatial attention modules. The approaches on rank 1, 3, 4, 7 and 8 use four modalities in their architectures (FLAIR, T1w, T2w, PD), whereas rank 5 does only use two of them (T1w, FLAIR). The architecture on rank 2 and 9 takes three modalities into consideration (FLAIR, T1w, T2w), like our approach that lands on rank 6.

Due to the fact that all the best performing approaches rely more or less on the U-Net architecture, the proposed architecture in this paper is also built based on this design. In contrast to 8 of the top 10 performing approaches, all of which are 3D^18–24,26^ or 2.5D^17^ based, we want to keep the computational cost low. Hence the focus lies on building a 2D image-based multi-modality CNN for MS lesion segmentation with distinct encoding paths for different modalities. This allows independent processing of the unique features of each modality. A more detailed rationale for this can be found in the Methods section.

Because of the fact that we don’t opt for a very deep neural network, nor do we deal with very large amounts of data, we also decided not to use ResNet blocks in our 2D approach, like Aslani et al.^25^, since they would have been more computationally intensive in general. In addition, we incorporated upsampling layers instead of transposed convolutional layers in the decoder of the U-Net to again achieve computational efficiency.

In summary, the contributions of the proposed architecture are threefold:

- We introduce a multi-input 2D-UNet for fast and precise segmentation of MS Lesions that can even be deployed on laptops without a GPU and still have usable inference duration times of about a minute, all while providing state-of-the-art performance.
- We implement a complementary encoding mechanism to have a better extraction of the features from every modality without weight sharing between the input channels that the network is able to learn modality-specific features without the inference of others. Moreover, we implemented a model selection strategy with a moving window that is not based solely on the validation loss. The network was trained on 2D-slices of all three orthogonal orientations from the 3D MRI volume to alleviate the strict locality of a 2D approach.
- We conducted extensive experiments on two publicly available datasets for MS Lesion detection regarding the segmentation performance and computational efficiency. Furthermore, an ablation study was performed to ensure that the design of our approach is well thought-out.

A preliminary version of this work appeared on TechRxiv^27^.

## Data and preprocessing

To evaluate the performance of the proposed method for MS lesion segmentation, two different publicly available datasets were used: the MICCAI 2016 MSSEG Lesion Segmentation Challenge dataset^28^ (denoted as the MSSEG dataset), and the ISBI 2015 Longitudinal MS Lesion Segmentation Challenge dataset (denoted as the ISBI dataset)^29^. These datasets are well researched^17–22,24–26^ and still relevant as they are also used in other current challenges, such as the Shifts 2.0 challenge^30^.

### ISBI 2015 longitudinal MS lesion segmentation challenge

The ISBI dataset consists of 19 subjects, acquired on a 3.0 T Philips MRI Scanner. Those subjects are divided into two separate sets, 5 of them are contained in the training set with the corresponding lesion masks and 14 subjects are in the test set, to which no ground truth is publicly available. For each subject, there are several acquisition time-points, ranging from 4 to 6. For each of those time-points, T1w, T2w, PDw and FLAIR image modalities were acquired. The scans are provided as raw- and preprocessed-versions^29^. The latter, which have been used in this study, are composed of 181 slices with a field-of-view (FOV)=181x217 and a 1 mm$^{3}$ voxel resolution. The segmentation performance of the proposed method for the test data set was evaluated by submitting the segmented binary masks to the challenge website^29,31^.

The ground-truth labels have been created mainly on the FLAIR images by two independent raters, using MIPAV^32^ (see figure A2). The comparison of both raters’ annotations is shown in Table 1.

### MICCAI MSSEG 2016 lesion segmentation challenge

The MSSEG Challenge was conducted at the Medical Image Computing & Computer Assisted Intervention (MICCAI) 2016 Conference. The dataset available for this work only consists of a training set, because the submission was already closed and so the test data was not available anymore. The training set includes both the pre-processed and the original images of 15 subjects. For every subject, there are seven lesion masks from independent raters and a consensus mask, which was created from the manual segmentation, using the logarithmic opinion pool based STAPLE algorithm^33^. The Dice-Sørensen-Coefficients (DSCs) of the independent raters compared to the consensus mask range from 0.68–0.77. The MRIs were acquired on three different scanners. One third of the subjects has been imaged on a 3 Tesla Siemens Verio Scanner. A 1.5 Tesla Siemens Aera Scanner has been used to acquire scans from another five of the subjects and the remaining ones were imaged with a 3 Tesla Philips Ingenia scanner. The provided images include 3D-FLAIR, 3D-T1w, 2D-PD/T2w and 3D-T1w-Gd (post contrast agent) images.

In this work, only the pre-processed FLAIR, T1-weighted and T2-weighted images are used for testing the architecture that was originally trained on the ISBI dataset. Due to the scanner variability (see Fig. A7), it serves as a good test for the robustness of the architecture.

## Methods

### Network architecture design

In this study, a 2D convolutional neural network, based on the U-Net^8^, is proposed. To exploit the MRI multi-modality analysis, we built an architecture with distinct parallel encoding channels for each modality. Multi-modal feature fusion blocks (MMFF) and multi-scale feature upsampling blocks (MSFU) were implemented to combine and up-sample the features from different modalities and different resolutions from the skip connections^25,34^. In the following sections, we first describe the preparation of the MRI data for machine learning applications. Next, the proposed network architecture and the training procedure is described in detail. Finally, we introduce the algorithm to choose the trained network for inference and the reconstruction of the 2D slices to a 3D MRI.

#### Preparation of the MRIs for machine learning applications

The provided architecture is built for two-dimensional data to get a good trade-off between performance and runtime. Therefore, the entire MRI volumes need to be sliced along axial, coronal and sagittal directions. This results in three 2D-representations of a given voxel and its surroundings in the corresponding plane. Later-on the distinct predictions can be combined to alleviate the downsides of the 2D-based approach^25^.

In case of the ISBI challenge data this would lead to differently sized slices for the orthogonal views, which cannot be presented simultaneously as inputs for the neural network due to their variable spatial resolutions.

Instead of just zero padding the smaller images, like Aslani et al.^25^, they have been simply resized, because even in slices with lesions the pixel distribution is heavily unbalanced either way and we did not want to reinforce this effect with even more background pixels. For the provided architectures, it is very convenient to work with inputs that have a resolution of $h\times w = 2^{n}\times 2^{n} \quad \text {pixels} ,\quad n\in {\mathbb {N}}$. To avoid losing information due to downsampling, all 2D input slices have been resized to a higher spatial dimension of $256\times 256$ pixels.

In the provided architectures, the *image data loader* from *Keras*^35^ was used to load and augment the slices in real time. All images were intensity normalized and the resulting slices were stored as .png files.

For the training set, only image slices which had at least one pixel labeled as lesion and, in addition, every 30th slice with no annotation have been chosen. This procedure assured a good trade-off between training speed and accuracy. Though the unlabeled slices are mostly seen as “unnecessary” information for the network^17,22,25^, their inclusion rendered the approach more robust. Moreover, the proposed architecture usually reached convergence about 3 to 8 times faster than other state-of-the-art approaches^17,19,25^ (see loss curves in Figure A1) , hence the drawbacks in training time due to the higher sample count could be easily accepted (see section Computational performance comparison to state-of-the-art approaches). For the validation and test set, every available slice was used to get a good grasp on how well the trained network will perform on whole brain MRIs.

#### Implementation details and training process of our multi-channel U-Net

**Figure 1:**
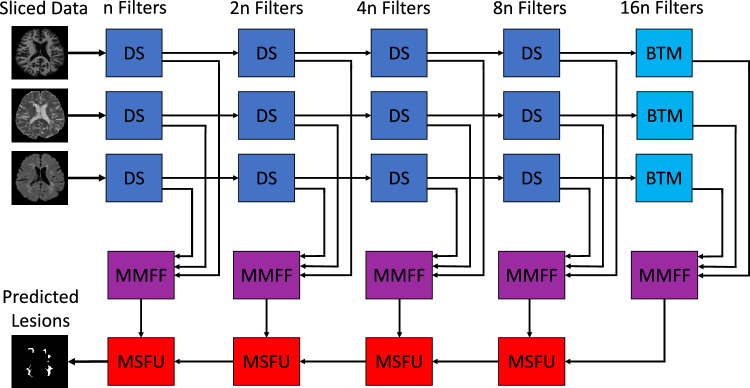
Proposed main architecture. This figure shows a schematic representation of the proposed main architecture. The prepared image slices are sequentially fed into the network with all its corresponding modalities. The network generates a prediction based on this current slice and then takes the next one. The first DS-Block has $n_0$ filters for the convolution layers. The filter count doubles for every DS-Block. The number of filters in the up-sampling branch matched the number of filters in the corresponding down-sampling blocks. The predictions were stacked for the axial, coronal and sagittal view which yielded three 3D volumes with their specific class membership probabilities. Those three volumes were then combined, averaged and finally thresholded to generate one binary volume as a prediction.

The proposed main architecture consists of three distinct encoding channels and one combined decoding path (see Fig. 1). Each of the encoding channels contains several down-sampling- (DS-) blocks and a bottom- (BTM-) block (see Fig. A3). The three input channels were implemented, because each of the three modalities has unique features, which in our opinion are best processed independently. By having separate branches, the network can learn modality-specific features without the interference of others, which might be crucial in case of complementary or even contradictory information about the tissues. As a CNN goes deeper, higher-level features are learned from the lower-level ones. For example, T1 images are very different from T2 or FLAIR images. In our opinion, mixing them from the beginning can lead to a confusing hierarchy of features that doesn’t effectively represent any of the modalities. With seperate branches, we ensure that the feature hierarchy remains relevant to the specific modalities, up to a certain point. Also, this has been demonstrated by Aslani et al.^25^, as well as in our ablation study in the experimental section. The first DS-block of every branch (with $n_{0}$ filters for the convolution layers) takes an original 2D-slice of the preprocessed volume from its corresponding modality as input and performs batch normalization, $3 \times 3$ convolutions and a $2 \times 2$ max-pooling operation. Accordingly, two separate outputs are generated in every block: (1) The output from the max-pooling layer is fed into the next DS-block and this process repeats, until the bottom layer is reached. For every step in the encoding stage, the filter count for convolutions doubles. (2) The output from the convolution layer of every DS-block also gets handed over as a skip connection to a Multi-Modal Feature-Fusion-Block (MMFF-block) (see Fig. A4*(left)*). There the features from the distinct channels (modalities) are concatenated and serve as high resolution input to the Multi-Scale Feature-Upsampling-Block (MSFU-block) (see Fig. A4*(right)*). For all convolutions, except for the last one, the ReLU activation function is used as non-linearity. In the output layer, it is the sigmoid activation function.

The low-resolution input to the MSFU-Block originates from the bottom- or another MSFU-Block. The output from the last multi-scale feature-up-sampling-block is fed into a $1 \times 1$ convolution layer with a sigmoidal activation function. This yielded a lesion probability for every pixel in the 2D image. This process gets repeated for all slices of the axial, coronal and sagittal views. All 2D images from a given view are then stacked to reconstruct a 3D volume. This leads to three entire 3D volumes, which then are averaged and finally thresholded to result in a binary output volume. The threshold was chosen in favor of the highest possible DSC.

This architecture was trained completely end-to-end with all three modalities and a batch-size of 15 for the training and 50 for the validation. The initial learning rate of 0.0003 was multiplied by 0.90 after every 300 batches. To avoid overfitting due to the small dataset, the images of the training set had to be heavily augmented. They were randomly rotated, flipped, shifted in width and height, zoomed and also were made darker and brighter. We also tried to include dropout layers in our network, but with them the segmentation performance was much worse (See Table 1). The filter count in the first DS-block was $n_{0}=32$, which led to $n_i =64, 128, 256, 512 ; i \in 2,3,4,5$ convolution filters of the subsequent DS blocks, respectively. In the up-sampling stage, the number of filters was identical to the down-sampling stages in reversed order. This led to a total number of 26, 242, 733 parameters, of which are 26, 213, 223 trainable- and 29, 510 non-trainable-parameters. The architecture with $n_{0}=32$ has been trained and evaluated in a leave-one-out-cross-validation (LOOCV), according to the protocol in table A1. It has also been fully trained and tested for all 20 combinations with $n_{0}=16$ and due to the excessive compute times, only on one dataset split combination with $n_{0}=64$, to estimate how the number of filters impacts the accuracy of the proposed method. In daily clinical practice, often some modalities on which the proposed architecture was originally trained on are not available. Because of this and to find out, which modalities are best suited for automated segmentation with a CNN, the main architecture was also trained and evaluated three times with only two channels and modalities (FLAIR & T1w, FLAIR & T2w, T1w & T2w) and three times with just one channel and one modality (only FLAIR or T1w or T2w).

The impact of the different modalities and the number of filters on the overall performance is illustrated in subsection Impact of filter count and different modalities on performance. As part of the ISBI Challenge, the primary architecture underwent training using a variety of loss functions. The model’s performance was assessed by submission of the predicted labels to the online evaluation system and further evaluated through cross-dataset validation. Transfer learning in terms of MRI scanner domain adaptation was employed, where we fine-tuned a pre-trained model initially trained on 2D MRI scans with a dataset comprising 3D scans from various scanner domains. Corresponding results are discussed in subsections Challenge submission, Cross dataset performance and Domain adaption.

All proposed architectures were implemented and tested in Python^36^ v.3.8.5 using Keras^35^ v.2.4.0 and Tensorflow^37^ v.2.4.1 backend. The training was performed on a machine with an NVIDIA RTX3090, an AMD Ryzen 9 3900X and 64 GB of RAM. The encoding channels and skip-connections were built based on the standard U-Net architecture^8^ with additional batch normalization. The up-sampling path for the multi-channel architectures was inspired by Aslani et al.^25^ but amended with additional batch normalization and changes to the up-sampling process. For all models, the adaptive moment estimation (ADAM) optimizer^38^ has been used. The weight initialization was done with the Glorot normal initializer (also referred to as Xavier normal initializer)^39^. An early stopping criterion with patience of 200 ensured that the training stopped, if the validation loss did not improve for 200 epochs. The loss calculation was performed batch-wise.

Given the heavy imbalance between foreground and background pixels in our dataset, there’s a high likelihood of forming batches with strong biases during training and validation. To mitigate this, we shuffle the training set after every epoch, preventing the model from learning the order of samples and protecting against potential bias from repeated exposure to the same sequence of images. While shuffling the training data is standard practice, we also opted to shuffle our validation set at the beginning of the evaluation process. Although it doesn’t directly enhance the model’s learning, this strategy aims to ensure that our batch-wise validation metrics are more stable and reliable, providing a better indication of how our network might perform on real-world data with similar characteristics. During training, not only the network with the “best” validation loss has been saved to disk, but also every third epoch in general. The shuffling could generate batches that jointly yielded a good overall loss in an early stage of the training, even though the network might have shown poor generalization (see Figure A1). Hence, it appeared that the network to be evaluated should not be chosen on the loss alone. Rather it should be also considered how stable the loss was in the given area in the training history. To pick the best network, an algorithm was developed, which is basically a moving window that computed the variance in loss over 50 epochs, and ordered these 50 neural networks based on their loss from the lowest to the highest. This calculation was applied to the last 150 epochs, where the window moved one epoch up after every calculation until the last window containing 50 epochs was reached. Subsequently, the algorithm selected the network with the lowest loss in the window of the smallest variance in the validation loss history. If this particular one had not been saved to disk the algorithm went to the next best choice and so on, until a good network had been found for evaluation.

#### Ablation study

We also performed an ablation study to ensure that the model architecture with all its building blocks is well designed and that its performance really is due to its structure. For this, we trained the architecture once without the MMFF-blocks (denoted as *first* in Table 2), once without the MSFU-blocks (denoted as *second* in Table 2) and once without both of them (denoted as *third* in Table 2). For better comparison, we also trained a standard 2D U-Net with only one input pattern encompassing three-channel gray scale images (one channel corresponding to one modality). Training and evaluation was done as detailed in table A1 with the same weight decay and initial learning rate as for the proposed model architecture.

#### Transfer learning

Transfer learning, specifically in terms of domain adaptation, was investigated to assess the adaptability of our architecture to MRI data from various scanner sites and different acquisition protocols, including 2D and 3D acquisition methods. The two best performing networks of the ISBI challenge, with regard to the DSC metric, were taken and fine-tuned on the MSSEG dataset, with a small initial learning rate of $10^{-5}$. For the exact training protocol refer to table A2. Every fifth epoch, the network was saved and every combination of the subjects was trained for 20 epochs. For the CNN with the filter count from 32 to 512 in the down- and up-sampling paths, this took about 45 minutes for a given combination of subsets. For the CNN with the corresponding filter count from 64 to 1024 the process took roughly $1.5\ h$.

### Metrics

For the performance evaluation, the same metrics as in the ISBI Challenge were chosen. Moreover, the exact same MATLAB scripts have been used, which are publicly available^40^. The metrics include the Dice-Sørensen-Coefficient^41,42^ (DSC), the Jaccard-Coefficient^43^, the Positive Prediction Value (PPV), the True Positive Rate (TPR), the Lesion-wise False Positive Rate (LFPR), the Lesion-wise True Positive Rate (LTPR), the Absolute Volume Difference (VD) and the Challenge Score (SC). Their definitions are given in the appendix for the convenience of the reader.

## Results and discussion

In the following section, the obtained results will directly be discussed in their relation to competitive models. Further arguments of a more general nature are also given to support our results.

### ISBI dataset results

#### Comparison of the main architecture with other state-of-the-art approaches

A nested leave-one-out cross-validation on annotated subjects was performed following the protocol in table A1. The results are illustrated in Table 1, where they are compared to other methods that were also trained on the ground truth annotations, provided by the first rater. We fully trained and evaluated the proposed architecture and the 3d fullres version of the nnUNet. The metrics for the other approaches are taken from Aslani et al.^25^. For the sake of comparison, the table only contains metrics, which are also provided for the other architectures. The minor differences in the inter-rater metrics, compared to the ones provided from Aslani et al.^25,44^ can be reproduced in our environment if we do not take the fifth acquisition from subject three into account. But for our calculations, all volumes have been considered. The nnUNet was trained for a reasonable number of 300 instead of the default 1000 epochs per combination of the LOOCV, since we deal with a very small dataset and the training durations are very high. No ensembled predictions and no test time augmentation (tta)^19^ were used here, for a fair comparison of the pure network’s performance.

**Table 1:** Results on the ISBI training set, compared to other state-of-the-art-methods.

Method	Rater 1			Rater 2		
	DSC	LTPR	LFPR	DSC	LTPR	LFPR
Rater 1	–	–	–	0.73 [0.69, 0.78]	0.65 [0.59, 0.72]	0.17 [0.11, 0.22]
Rater 2	0.73 [0.69, 0.78]	0.83 0.78, 0.89]	0.35 [0.28, 0.41]	–	–	–
Jesson et al.^45^	0.7040	0.6111	*0.1355*	0.6810	0.5010	*0.1270*
Maier & Handels (GT1)^46^	0.7000	0.5333	0.4888	0.6555	0.3777	0.4444
Brosch et al. (GT1)^15^	0.6844	0.7455	0.5455	0.6444	0.6333	0.5288
Aslani et al. (GT1)^44^	0.6980	*0.7460*	0.4820	0.6510	*0.6410*	0.4506
Aslani et al. (GT1)^25^	*0.7649*	0.6697	**0.1202**	*0.6989*	0.5356	**0.1227**
Isensee et al. - nnUNet (GT1)^19^	0.73 [0.72, 0.74]	0.67 [0.65, 0.69]	0.19 [0.17, 0.15]	0.67 [0.66, 0.68]	0.54 [0.52, 0.56]	0.18 [0.17, 0.20]
Proposed Architecture with Dropouts ($n_{0}$ =32) (GT1)	0.72 [0.70, 0.75]	0.66 [0.62, 0.70]	0.32 [0.28, 0.36]	0.66 [0.64, 0.69]	0.55 [0.51, 0.59]	0.35 [0.31, 0.40]
Proposed Architecture ($n_{0}$=32) (GT1)	**0.78** [0.76, 0.79]	**0.75** [0.71, 0.78]	0.27 [0.25, 0.29]	**0.72** [0.70, 0.74]	**0.64** [0.60, 0.67]	0.28 [0.24, 0.30]

This table compares results from the proposed main architecture, with other state-of-the-art approaches on the ISBI dataset. All methods were trained based on annotations from rater 1^16^. The mean values of *DSC*, *LTPR* and *LFPR* are shown. Where available, the 95%-confidence intervals are denoted in square brackets. Bold and italic values refer to the first- and second-best method of the corresponding metrics, respectively.

The proposed architecture yields the best results regarding the DSC and the LTPR metrics. The LFPR ranks only third. In other words, the tested approach delineates the lesions most precisely and also finds the most of them, but also misclassifies more than^25,45^ and^19^. An interesting observation is that the nnUNet did not perform well in our LOOCV, regarding the DSC and LTPR. One reason for this could be that for the fair comparison we tested the pure network’s performance without ensembling and additional postprocessing on which the nnUNet usually relies. However, due to its 3D nature, the LFPR is still much better than for the proposed method. In general, FPs are one major drawback of any 2D approach without additional postprocessing. In brain MRIs, the protrusions of the cortex can look like lesions, but after inspecting adjacent slices one can easily identify such protrusions as healthy tissue. However, 2D architectures are unable to merge the information from adjacent slices to the one that is currently being segmented. These specific FPs could be eliminated by removing all segmented lesions that were only two dimensional. Alternatively, an additional 3D CNN could be deployed to only processes the areas that were classified as a lesion in the 2D architecture^19^. However, it appears that the overall metrics of the proposed architecture are well suited for clinical practice, because medical experts still are the last instance in deciding whether any segmented patch represents a lesion or not. Furthermore, considering the volumes segmented with the proposed approach, it appears more likely to draw attention to lesions, which the physician otherwise might have missed. Examples of predictions from brains with either a heavy or a low lesion load are shown in Fig. A5.

The prediction time for a whole MRI volume is about four seconds on our GPU. A more in-depth performance comparison with other methods can be found in section Computational performance comparison to state-of-the-art approaches.

#### Results of an ablation study

For the first test, we just replaced the MMFF-blocks with a concatenation layer for the three inputs. In the second test, we replaced the MSFU-blocks with a 2D-Convolution followed by an upsampling layer for the low resolution input and a concatenation layer to combine the upsampled low-resolution and the high-resolution input (see Fig. A4). For the third test, we subsequently made both changes at the same time. The results are illustrated in Table 2.

**Table 2:** Results of the ablation study, including comparison to a standard U-Net.

Modalities	Ground Truth Rater 1						
	DSC	Jaccard	PPV	TPR	LFPR	LTPR	VD
Baseline	**0.78** [0.76, 0.79]	**0.64** [0.62, 0.66]	**0.81** [0.79, 0.82]	**0.76** [0.74, 0.79]	**0.27** [0.25, 0.30]	*0.74* [0.71, 0.78]	**0.13** [0.10, 0.16]
First	*0.69* [0.67, 0.71]	0.53 [0.51, 0.55]	0.71 [0.69, 0.73]	*0.70* [0.67, 0.73]	0.36 [0.33, 0.40]	0.73 [0.69, 0.77]	*0.23* [0.19, 0.27]
Second	0.67 [0.65, 0.69]	0.51 [0.49, 0.53]	0.71 [0.69, 0.74]	0.68 [0.65, 0.72]	0.42 [0.39, 0.45]	0.74 [0.70, 0.78]	0.28 [0.24, 0.33]
Third	0.68 [0.66, 0.70]	0.52 [0.50, 0.54]	*0.72* [0.69, 0.74]	0.69 [0.66, 0.72]	0.43 [0.40, 0.47]	**0.75** [0.70, 0.78]	0.27 [0.23, 0.31]
Standard U-Net	0.67 [0.65, 0.69]	0.51 [0.49, 0.53]	0.70 [0.68, 0.73]	0.68 [0.65, 0.72]	*0.35* [0.31, 0.39]	0.72 [0.68, 0.77]	0.25 [0.21, 0.30]

This table illustrates the performance comparison of the proposed architecture (denoted as *baseline*) to the architecture without the MMFF-/MSFU-blocks. First corresponds to the results without the MMFF-blocks. Second corresponds to the performance excluding the MSFU-blocks. Third corresponds to the results without both building blocks. The standard U-Net is a multichannel implementation with only one input, where every distinct channel corresponds to a MRI modality. The best and second-best results are written in bold and italic, respectively. For all metrics, the 95%-confidence intervals are given in square brackets.

Leaving out one or more of the building blocks from our architecture lead to a much worse performance in our tests, than our proposed approach. The multichannel CNN with all its building blocks had the best performance for all metrics, except for the LTPR. However, within the given uncertainty measure, all LTPR results agree with each other. The results with the standard U-Net were the worst for almost every metric. Overall, this confirms the well thought out design choices and also emphasizes the superiority of our approach as a whole compared to a standard U-Net implementation that uses weight sharing between the input channels for all modalities.

#### Impact of filter count and different modalities on performance

The results from the architectures with different amounts of filters, are illustrated in table A3. The training time for one combination of the LOOCV for the filter sets of $n_{0}=16$, $n_{0}=32$ and $n_{0}=64$ was ~2.5*h*, 4.2*h* and 7.0*h*, respectively. The network had the highest DSC and lowest LFPR with the $n_{0}=32$ filter set. Though the LTPR was a little less than in the architecture with $n_{0}=16$, the results in terms of DSC and LFPR metrics were much better. Compared to the annotations of rater 1, the results with the biggest filter set, trained on its annotations, were worse than with the medium sized filter set. The LTPR and LFPR metrics were better if compared to the annotations of rater 2. But, due to better DSC metrics achieved with the medium sized filter set, and the pronounced increase in training time for $n_{0}=64$, all further tests were performed with $n_{0}=32$.

The segmentation results for the networks with different modalities are summarized in Table 3.

**Table 3:** Results of the main architecture with one, two and three branches and modalities.

Modalities	Ground Truth Rater 1						
	DSC	Jaccard	PPV	TPR	LFPR	LTPR	VD
FLAIR, T1w, T2w	*0.78* [0.76, 0.79]	*0.64* [0.62, 0.66]	*0.81* [0.79, 0.82]	**0.76** [0.74, 0.79]	*0.27* [0.25, 0.30]	**0.74** [0.71, 0.78]	*0.13* [0.10, 0.16]
FLAIR, T2w	**0.78** [0.77, 0.80]	**0.65** [0.63, 0.66]	**0.81** [0.79, 0.82]	*0.77* [0.75, 0.79]	**0.27** [0.24, 0.29]	*0.74* [0.70, 0.78]	**0.13** [0.10, 0.15]
FLAIR, T1w	0.75 [0.73, 0.77]	0.61 [0.59, 0.64]	0.86 [0.84, 0.88]	0.69 [0.66, 0.72]	0.23 [0.20, 0.25]	0.72 [0.68, 0.75]	0.19 [0.15, 0.23]
T1w, T2w	0.65 [0.64, 0.67]	0.49 [0.47, 0.51]	0.71 [0.69, 0.73]	0.62 [0.60, 0.65]	0.39 [0.35, 0.42]	0.65 [0.61, 0.68]	0.18 [0.15, 0.21]
FLAIR	0.75 [0.74, 0.77]	0.61 [0.59, 0.63]	0.81 [0.79, 0.83]	0.72 [0.70, 0.74]	0.34 [0.30, 0.37]	0.70 [0.66, 0.74]	0.16 [0.12, 0.19]
T2w	0.65 [0.63, 0.67]	0.49 [0.47, 0.50]	0.69 [0.66, 0.72]	0.64 [0.61, 0.66]	0.45 [0.41, 0.49]	0.63 [0.60, 0.67]	0.22 [0.20, 0.25]
T1w	0.48 [0.45, 0.50]	0.32 [0.30, 0.35]	0.78 [0.76, 0.80]	0.36 [0.33, 0.38]	0.30 [0.27, 0.32]	0.52 [0.49, 0.56]	0.54 [0.51, 0.57]

This table contains all metrics that were achieved by the two- and one-branch architectures compared with the initial configuration with three branches. The best and second-best results are written in bold and italic, respectively. For all metrics, the 95%-confidence intervals are given in the square brackets.

An interesting observation was the fact that the 2-channel architecture, trained on FLAIR and T2w images, yielded a better performance in all metrics than the 3-channel version, except for the TPR and LTPR metrics. However, the difference to the latter was very small. The reason for the improvement compared to the three modality version could be the so-called T1 black holes (see Fig. A6), which only appear for certain chronic lesions, while all of the lesions are always visible in the FLAIR and T2w images. Also, the images mainly have been labeled on the FLAIR modality. This combination probably disturbed the neural network and rendered its training more unstable.

#### Challenge submission

Due to the fact that a whole run of all 20 combinations from the training protocol took much compute time, the fine tuning was evaluated by multiple challenge submissions. The networks were trained on the FLAIR, T1w and T2w modalities according to the protocol in table A4. Those networks were also used for cross-dataset evaluation and transfer learning (see subsections Cross dataset performance and Domain transfer). The architectures with the best results in the cross-dataset evaluation were used for challenge submission. These results are shown in table A5.

According to those metrics, the main architecture with a filter set of $n_{0}=32$ yielded the best TPR and DSC metrics, but was only ranked third regarding the submission score, compared with the other model configurations. The best submission score of $SC = 92.661$ was achieved by the main architecture with the medium sized filter set, trained with the combined dice and binary-cross-entropy loss functions. However, according to the ISBI Challenge^16^, obtaining a submission score of $SC \ge 90$ with an automated segmentation approach means that the method performs similar to a human expert. The comparison with other state-of-the-art published results for the ISBI challenge placed the proposed architecture in sixth place. With this, the approach was ahead of all other 2D-methods and even better than three of the top ten three dimensional architectures. These results are presented in Table 4.

**Table 4:** Performance comparison with state-of-the-art architectures in the ISBI challenge.

Approach	Modalities	CNN type	DSC	PPV	TPR	LFPR	LTPR	Submission score
2.5D Tiramisu^17^	FLAIR, T1w, T2w, PD	2.5D	0.64	*0.91*	0.53	*0.12*	0.52	**93.358**
ALL-NET^18^	FLAIR, T1w, T2w	3D	0.63	0.91	–	0.12	0.533	93.32
nnUNet^19^	FLAIR, T1w, T2w, PD	3D cascade	**0.69**	0.85	*0.61*	0.17	*0.55*	*93.09*
nnUNet^20^	FLAIR, T1w, T2w, PD	3D	*0.68*	0.86	0.60	0.16	0.54	93.03
DeepLesionBrain^21^	FLAIR & T1w	3D	0.65	0.89	0.55	0.13	0.49	92.85
Multi-branch U-Net (proposed)	FLAIR, T1w, T2w	2D	0.64	0.85	0.56	0.20	*0.55*	92.661
IMAGINE^22^	FLAIR, T1w, T2w, PD	3D	0.58	**0.92**	0.46	**0.09**	0.41	92.49
Self-adaptive network ^24^	FLAIR. T1w, T2w, PD	3D	*0.68*	0.78	**0.65**	0.27	**0.60**	92.41
Multi-branch ResNet ^25^	FLAIR, T1w, T2w	2D	0.61	0.90	0.49	0.14	0.41	92.12
Attention-Based CNN ^26^	FLAIR, T1w	3D	0.64	–	–	0.39	0.45	–

This table compares the performance metrics of state-of-the-art published architectures with the proposed architecture. The proposed method lands on rank 6, where it is able to outperform all 2D approaches and even two of the state-of-the-art 3D approaches, regarding the submission score. The best and second-best results are written in bold and italic, respectively.

The results were even more impressive if one keeps in mind that the proposed method did not use all of the available modalities, like the architectures ranked 1, 3, 4, 7 and 8. Moreover, the score has been achieved by just one network predicting labels on the test set and not ensembling multiple predictions for the submission, as the other approaches did.

#### Computational performance comparison to state-of-the-art approaches

The proposed approach provides state-of-the-art results, while still being computationally light. To emphasize this, we performed several tests on our machine with the RTX3090, as well as on a 2021 MacBook Pro with a M1 Pro CPU and 16GB RAM. For comparison, we chose the nnUNet^19,20^, since it is publicly available, easy to deploy and reached rank 3 and 4 with two different configurations in the ISBI challenge. The 2.5D Tiramisu CNN^17^, which reached first place in the ISBI challenge is also publicly available, easy to use and served as a third comparison for our approach. The results of these tests are shown in Table 5.

**Table 5:** Execution times of training and inference on GPU and CPU.

Approach	Inference GPU	Inference CPU	Train one fold	GFLOPs	RAM	VRAM
2.5D Tiramisu ^17^	*7 s*	*5.5min*	*12.5 h*	23.9	6 GB	19.3 GB
nnU-Net^19^	15 s (5 s)	207 min (5.2 min)	34.7 h (10.4 h)	2.74 e+03	22 GB	9.2 GB
nnU-Net small^20^	12 s (4 s)	164 min (4.2 min)	29.4 h (9.2 h)	2.21 e+03	22 GB	8.9 GB
Multi-branch U-Net (proposed)	**4 s**	**67 s**	**4.2 h**	53.2	4GB	9.4 GB

This table compares the duration times of two other publicly available state-of-the-art architectures on our systems, as well as the performance metrics from the ISBI challenge submission. The best and second-best results are written in bold and italic, respectively.

Compared to the 2.5D Tiramisu, we have an acceleration on the GPU of the factor 1.75 and on the MacBook we have a 4.9 times faster inference. Training the 2.5D Tiramisu model with the same input size as our approach for one of our 20 combinations from our training protocol (Table A1) took 12.5 h on our machine, whereas our architecture just required 4.2 h of training. For all 20 combinations, this would result in a total training time of 10.4 days for the 2.5D Tiramisu CNN, compared to 3.5 days with our architecture. The speedup compared to the 3D nnUNet variants is even more drastic. The inference for our architecture on the GPU is 3 to 3.8 times faster than the nnUNet. On the MacBook, the inference is extremely slow with 164 to 207 minutes for one prediction. This can be accelerated to an inference duration time of only 4 to 5 min, if ensembled predictions and test time augmentation are turned off, but that reduced the segmentation performance in our tests by about 5 percent, regarding the DSC and the results from the ISBI challenge were most probably done with default settings of the framework. However, even if we speed up the predictions and accept worse segmentation results than with our approach, the acceleration of our architecture lies at factor 3.8 to 4.7. If one wants to have the same segmentation performance that was shown in the ISBI challenge, the nnUNet is 147 to 185 times slower with its 164, respectively 207 min of duration for inference on our MacBook, than our proposed approach.It is a basic design choice of the nnU-Net framework to train the network for a total number of 1000 epochs, where each epoch is defined as 250 batches, no matter how large or small the batches are. Also the network does always use the model from the last trained epoch for the inference^19^. However, for a comparison with the training durations of our network, we provided the training durations for 300 epochs in round brackets, too. Training one of our 20 combinations from the LOOCV would take 29.4 to 34.7 h for a full run of 1000 epochs. Doing this with all the combinations, this would result in 24.5 to 29 days of training, compared to 3.5 days with the proposed approach. For the 300 epoch variants, this would take 7.7 to 8.7 days, which still is a factor of 2.2 to 2.5. In terms of floating point operations (FLOPs) for one forward pass through the architecture, without taking data preprocessing and postprocessing into account, the 2.5D Tiramisu network with 23.9 GFLOPs is the lightest in this comparison. The proposed method with its 53.2 GLFLOPs performs about 2.2 times the amount of FLOPs than the 2.5D Tiramisu CNN, but is still faster in inference. The nnU-Net clearly has the highest computational requirements in our comparison with its 2.21 to 2.47 TFLOPs, due to its 3D nature, hence in theory one forward run is about 41 to 46 times more computational intensive than our approach. However, this doesn’t really reflect as drastic in the inference times. During training, the nnU-Net has the highest RAM utilization of 22 GB and our approach is the least demanding. with just 4 GB. Regarding the VRAM, the 2.5 D Tiramisu has the highest utilization of 19.3 GB. The nn-UNet and our approach are in the same range with about 9 GB.

Compared to the 2.5D Tiramisu, our architecture shows impressive speedup, both on the GPU and on the MacBook, being significantly more efficient in terms of training time. When compared to the 3D nnUNet, the speedups of our architecture are even more pronounced, being 3 to 3.8 times faster in inference on the MacBook, due to the more complex data processing required for the nnUNet. Although the nnUNet is significantly more intensive in terms of FLOPs due to its 3D nature, this is not reflected as drastically in the inference times. It is also surprising that despite the higher FLOP count of our method compared to the 2.5D Tiramisu, our architecture is still faster in inference. This could be due to the more complex data preparation requirements needed for the 2.5D model. Overall, our architecture provides a substantial increase in efficiency over both compared models.

### MICCAI MSSEG dataset results

#### Cross dataset performance

To test how good the architecture would perform on another dataset, acquired on other scanners and with different pre-processing, the neural networks were trained according to the protocol in table A4. The networks for those tests were the ones used for the ISBI Challenge submission. Different numbers of filters in the encoder-decoder paths and several loss functions were tested. The best results from every network configuration are presented in table A6. This shows the good cross-dataset performance of our architecture, trained on the ISBI dataset and evaluated on the MSSEG dataset. The outcomes are even more remarkable, considering that the networks were trained using 2D scans from the ISBI dataset. Yet, when measured by DSC, PPV, TPR, and VD, within the context of confidence intervals, their performance was on par with several of the raters relative to the consensus mask (refer to table A7), even on 3D data of superior resolution. This is noteworthy, especially since the rater’s annotations invariably contribute to the formation of the consensus mask. However, the method falls short concerning the LFPR, attributable to its 2D nature and the absence of any additional post-processing. The cross-dataset performance was also evaluated against other approaches. For comparison, we took the metrics provided by Kamraoui et al.^21^ The results are shown in Table 6.

**Table 6:** Results of cross-dataset evaluation compared to other approaches.

Approach	Consensus mask				
	DSC	PPV	TPR	LFPR	LTPR
Multi-branch U-Net (proposed)	**0.68** [0.65, 0.71]	**0.77** [0.73, 0.81]	*0.63* [0.59, 0.67]	0.63 [0.58, 0.68]	0.64 [0.60, 0.68]
DeepLesionBrain^21^	0.639	*0.768*	0.608	*0.319*	**0.700**
2.5D Tiramisu^17^	*0.664*	0.741	**0.658**	**0.284**	*0.695*

This table compares the results for the cross-dataset performance from our approach to other state-of-the-art approaches. All networks were initially trained on the ISBI set^16^ and tested on the MSSEG dataset^28^. The numbers are averaged over all subjects. Our approach is using three modalities (FLAIR, T1w, T2w), whereas the other two were trained with two modalities (FLAIR, T1w). The numbers in square brackets indicate the 95%-confidence intervals. The performance metrics for the other approaches were taken from R. A. Kamraoui et. al.^21^. The best and second-best results are written in bold and italic, respectively.

Furthermore, our approach surpasses both the 2.5D Tiramisu^17^ and DeepLesionBrain^21^ in terms of DSC and PPV metrics, demonstrating not only a significant overlap with the ground truth lesions but also the highest proportion of true positives among the predicted positives in the training dataset. Despite these strengths, there are downfalls because of the models 2D nature. It exhibits a higher lesion-wise false-positive rate compared to the other methods, signaling an opportunity for further enhancement for incorporating post-processing methods, particularly in minimizing over-segmentation or incorrect lesion detection. In terms of the TPR metric, the performances of the different approaches are comparable, falling within the same confidence intervals. Collectively, these findings suggest that the proposed model performs well at generalizing across datasets.

#### Domain transfer

Transfer learning in terms of domain transfer was investigated to test the ability of the proposed segmentation model to be quickly and easily tailored to different scanner domains. Table A8 compares the results of two pre-trained architectures. They were trained on the ISBI dataset and fine tuned with the MSSEG dataset. The evaluation was done based on every fifth epoch.

**Figure 2:**
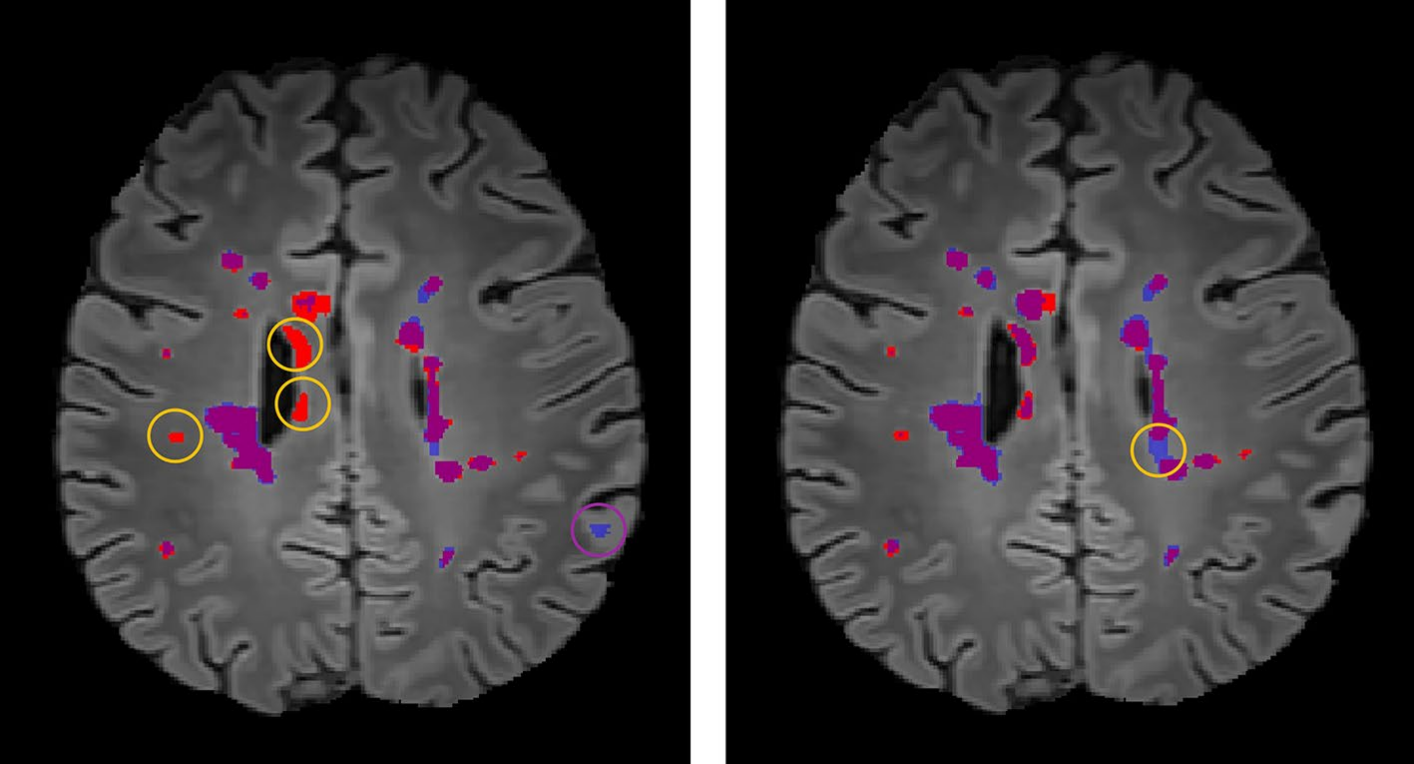
Comparison of a segmentation before and after domain adaption. This figure illustrates the segmentation of subject *08002CHJE* from the MSSEG dataset^28^ before (**a**) and after (**b**) the transfer learning. In both images, the ground truth is red, the segmented lesions are in blue and purple areas indicate an overlap of both. In (**a**), the purple circle shows an FP lesion and the yellow circles highlight FNs. In (**b**), neither the FP lesion nor the FNs were detected anymore. The lesions around the yellow circle in (**b**) were just not distinguished and classified as one.

In Fig. 2, the segmentation achieved with fine tuning after 10 epochs corroborates its superior performance over the baseline segmentation of the cross dataset evaluation. The results even allowed an increase of the binarification threshold by $10\ \%$. Furthermore, as the FNs from the baseline segmentation disappeared completely in this slice, the network was much more certain about its decision, whether a segmented patch is a lesion or not. Fig. 2 also shows that the lesions were delineated much more precisely after transfer learning than before. Considering cross-dataset evaluation, the baseline tests of the larger filter set yielded the best results. However, during domain adaption, the performance increase was less impressive, and, in fact, the smaller filter set produced the better results. For both models, the best DSC has been accomplished after 10 epochs of transfer learning. Overall, adaption to different scanner domains worked very well on the proposed architecture ($n_{0}=32$) with an additional fine-tuning of $5-10$ epochs employing the MSSEG dataset. Also, a very low computational demand rendered this approach very practical.

## Conclusion

The primary goal of this work was to develop and implement a fast and accurate automated deep learning model for detection and segmentation of multiple sclerosis lesions in brain MRI images. To do so, a 2D U-Net-like CNN architecture with three distinct input channels, corresponding to three different MRI modalities, was designed. The model is basically a combination of several existing approaches with appropriate modifications and extensions to lower the computational complexity. For the proposed model, a combination of dice- and binary-cross-entropy losses provided best results. When different loss functions were deployed to train the proposed architecture, a simple $L_2$-loss yielded the worst results in this study. This differs from Zhang et al.^17^, who achieved their best score with it. Also, contrary to Feng et al.^24^ and Ibtehaz et al.^47^, a further increase of the base model’s performance could not be achieved by implementing dropout layers or ResNet connections, though the related architectures were rather similar. Another interesting observation was that the learning process probably got distracted to some extent by the T1w images.

Concerning cross-dataset evaluation, our architecture outperformed the competing state-of-the-art approaches regarding the metrics DSC and PPV. In other words, the network was able to most precisely segment the lesions in the cross dataset. Moreover, most of the voxels that have been predicted as a lesion were correctly identified. Subsequently, the transfer learning yielded results that were comparable to the human raters of the challenge.

Overall, the metrics in Table 1—including the $95\ \%$-confidence intervals—are comparable to the inter-rater performance. We reached a challenge submission score of $SC = 92.67\ \%$, which renders the segmentation performance comparable to a human expert^16^. The architecture also achieved a cross-dataset performance that is comparable to human raters and transfer learning in terms of scanner domain adaption worked well. Thus it was possible to show that a proper architecture, well trained on one dataset could be easily and quickly adapted to images of another scanner, even with just a few samples available for fine-tuning. We were able to demonstrate a significant speedup in terms of training durations and inference times, compared to three of the top 10 approaches in the ISBI challenge. These results demonstrate the efficiency of the proposed architecture, while still providing state-of-the-art performance.

The proposed model ranked sixth on the ISBI challenge leaderboard. Accordingly only approaches, which were reported in open-access scientific journals were considered for performance comparisons. In summary, the proposed model was superior to all 2D-methods and also some 3D approaches published in the challenge, although the latter are computationally much more expensive as we could demonstrate and they used more modalities than were used in this study.

### Supplementary Information

Supplementary Information.

## Data Availability

The full code implementation, including instructions for deployment will be publicly available at https://github.com/Nanex101195/Multibranch-2D-UNet after publication. The challenge datasets used for evaluating our architecture are available at the challenge websites^31, 48^.
