# Supplementary material for: Investigation of an efficient multi-modal convolutional neural network for multiple sclerosis lesion detection
Source: Sci Rep. 2023 Nov 30;13:21154. doi: 10.1038/s41598-023-48578-4 (PMC10689724; doi:10.1038/s41598-023-48578-4)
Supplement: Supplementary file 1 — Supplementary Information. [file 41598_2023_48578_MOESM1_ESM.pdf]

Investigation of an efficient Multi-Modal Convolutional Neural Network for Multiple Sclerosis Lesion Detection

Florian Raab[1,*], Wilhelm Malloni[2], Simon Wein[1,2], Mark W. Greenlee[2], Elmar W. Lang[1]
[1]University of Regensburg, Computational Intelligence and Machine Learning Group, 93051 Regensburg, Germany
[2]University of Regensburg, Experimental Psychology, 93051 Regensburg, Germany

Supplementary Information

The definitions of all used metrics are given here for the convenience of the reader. In the following, TP denotes the true positives, FP the false positives, TN the true negatives and the false negatives are denoted as FN. These terms refer to the correct/incorrect identification of a given voxel as belonging to a lesion or not.

$$DSC = \frac{2TP}{FN+FP+2TP}; \qquad Jaccard = \frac{TP}{TP+FP+FN}. \tag{1}$$

The coefficients are in a range between 0 and 1, where 1 indicates a complete overlap of all voxels from the predicted labels with those of the true mask. The only difference between both of them is that the true positives are twice as important in the DSC, compared to the Jaccard.

$$PPV = \frac{TP}{TP+FP}; \qquad TPR = \frac{TP}{TP+FN}. \tag{2}$$

The PPV is a measure of how many voxels that were classified as lesions were correct. The TPR is a metric that indicates how many of the voxels that should have been predicted were missed.

$$LFPR = \frac{LFP}{PL}; \qquad LTPR = \frac{LTP}{RL}. \tag{3}$$

Here, LFP denotes the number of lesions in the prediction that do not overlap with a lesion in the reference segmentation (at least one pixel) and PL is the total number of lesions in the predicted volume. The LTP is a metric for the number of lesions in the output segmentation that overlap with a lesion in the reference segmentation and RL is the total number of lesions in the reference segmentation. These are important metrics, since the others do not take small lesions that much into account. For those both metrics, a small lesion is as important, as a big one.

$$VD = \frac{\left|Vol_{pred}^{lesion} - Vol_{true}^{lesion}\right|}{Vol_{true}^{lesion}} = \frac{\left|(TP+FP) - Vol_{true}^{lesion}\right|}{Vol_{true}^{lesion}}. \tag{4}$$

There, Vol_{true}^{lesion} denotes the total lesion volume in the reference segmentation and Vol_{pred}^{lesion} subsequently is the total lesion volume in the predicted segmentation.

$$SC = \frac{1}{|R|\cdot|S|}\cdot\sum_{R,S}\left(\frac{DSC}{8}+\frac{PPV}{8}+\frac{1-LFPR}{4}+\frac{LTPR}{4}+\frac{Cor}{4}\right), \tag{5}$$

Here, R denotes the set of all raters, S the set of all subjects and Cor is the Pearson's correlation coefficient [1] of the volumes. By reaching a score of 90 or higher in the ISBI challenge, the algorithm is said to be comparable to an expert human rater. [2]

Table A1. Evaluation protocol with ISBI 2015 challenge training data. This table illustrates the evaluation protocol with the ISBI 2015 challenge [2] training data. A nested leave-one-subject-out-cross-validation (3 subjects for training, one for validation and one for testing) was implemented for non-biased evaluation. The data were split subject wise to simulate real-world conditions and the numbers denote the given subject with all its corresponding time points.

Training	Validation	Test
1, 2, 3	4	5
1, 2, 4	3	5
1, 3, 4	2	5
2, 3, 4	1	5
1, 2, 3	5	4
1, 2, 5	3	4
1, 3, 5	2	4
2, 3, 5	1	4
1, 2, 4	5	3
1, 2, 5	4	3
1, 4, 5	2	3
2, 4, 5	1	3
1, 3, 4	5	2
1, 3, 5	4	2
1, 4, 5	3	2
3, 4, 5	1	2
2, 3, 4	5	1
2, 3, 5	4	1
2, 4, 5	3	1
3, 4, 5	2	1

Table A2. Transfer learning protocol. This tables illustrates the transfer-learning training protocol on the MSSEG [3] dataset with a pretrained CNN that was initially trained on the ISBI training dataset [2]. **(a)** Lists which subjects the several subsets included and **(b)** illustrates the leave-one-out cross-validation process. The subsets have been formed in a way that in every subset there is at least one subject from each scanner.

(a)

Subset	Subjects
First	01016, 07001, 08002
Second	01038, 07003, 08027
Third	01039, 07010, 08029
Fourth	01040, 07040, 08031
Fifth	01042, 07043, 08037

(b)

Training	Validation	Test
First, Second, Third	Fourth	Fifth
First, Second, Fourth	Third	Fifth
First, Third, Fourth	Second	Fifth
Second, Third, Fourth	First	Fifth
First, Second, Third	Fifth	Fourth
First, Second, Fifth	Third	Fourth
First, Third, Fifth	Second	Fourth
Second, Third, Fifth	First	Fourth
First, Second, Fourth	Fifth	Third
First, Second, Fifth	Fourth	Third
First, Fourth, Fifth	Second	Third
Second, Fourth, Fifth	First	Third
First, Third, Fourth	Fifth	Second
First, Third, Fifth	Fourth	Second
First, Fourth, Fifth	Third	Second
Third, Fourth, Fifth	First	Second
Second, Third, Fourth	Fifth	First
Second, Third, Fifth	Fourth	First
Second, Fourth, Fifth	Third	First
Third, Fourth, Fifth	Second	First

Table A3. Comparison of different filter sets in the main architecture. The table on top compares results obtained with the proposed main architecture with two different filter sets, evaluated according to the protocol in table A1. The second table compares three different filter sets used in the main architecture, based on the evaluation from the first combination of the training protocol described in table A1. GT denotes the ground truth on which the corresponding architecture has been trained on. The best and second-best results are written in bold and italic, respectively. For all metrics, the 95%-confidence intervals are given in the square brackets written in blue.

	Rater 1			Rater 2		
**Amount of Filters**	**DSC**	**LTPR**	**LFPR**	**DSC**	**LTPR**	**LFPR**
**Rater 1**	-	-	-	0.73 [0.69, 0.78]	0.65 [0.59, 0.72]	0.17 [0.11, 0.22]
**Rater 2**	0.73 [0.69, 0.78]	0.83 [0.78, 0.89]	0.35 [0.28, 0.41]	-	-	-
**Main,** ($n_0 = 16$) **(GT1)**	*0.76* [0.74, 0.77]	0.71 [0.68, 0.75]	*0.31* [0.27, 0.34]	*0.70* [0.68, 0.72]	0.60 [0.57, 0.64]	0.35 [0.30, 0.39]
**Main,** ($n_0 = 16$) **(GT2)**	0.74 [0.67, 0.72]	**0.77** [0.73, 0.80]	0.39 [0.36, 0.42]	0.69 [0.67, 0.72]	**0.69** [0.66, 0.72]	*0.36* [0.32, 0.39]
($n_0 = 32$) **(GT1)**	**0.78** [0.76, 0.79]	0.75 [0.71, 0.78]	**0.27** [0.25, 0.29]	**0.72** [0.70, 0.74]	0.64 [0.60, 0.67]	0.28 [0.24, 0.30]

Comparison of different filter sets based on the first combination in A1.

	Rater 1			Rater 2		
**Amount of Filters**	**DSC**	**LTPR**	**LFPR**	**DSC**	**LTPR**	**LFPR**
**Main,** ($n_0 = 16$) **(GT1)**	*0.73* [0.66, 0.80]	*0.86* [0.69, 1.00]	0.39 [0.07, 0.71]	0.69 [0.61, 0.77]	0.63 [0.40, 0.85]	0.35 [0.01, 0.68]
**Main,** ($n_0 = 16$) **(GT2)**	0.72 [0.64, 0.80]	*0.87* [0.67, 1.00]	0.32 [0.23, 0.41]	0.69 [0.61, 0.77]	**0.65** [0.27, 0.34]	0.32 [0.23, 0.41]
$n_0 = 32$ **(GT1)**	**0.74** [0.68, 0.80]	**0.87** [0.71, 1.00]	**0.27** [0.15, 0.38]	**0.70** [0.62, 0.77]	0.64 [0.44, 0.84]	*0.29* [0.44, 0.42]
$n_0 = 64$ **(GT1)**	0.73 [0.68, 0.78]	0.86 [0.70, 1.00]	*0.31* [0.03, 0.59]	*0.69* [0.66, 0.73]	*0.67* [0.52 , 0.89]	**0.29** [0.00, 0.67]

Table A4. Choice of datasets for training the networks, used for challenge submission. This table illustrates the choice of the datasets used in the training process for the ISBI challenge submission and the transfer learning.[2] The data split is acquisition-time point wise to have the most possible variation in training samples and the most possible generalization. For the training set, all subjects were used and one time point of every subject has been reserved for the validation set. Due to the variation in acquired time points, for the first combination the last acquisition of every subject was used in the validation set and in the other combinations, the same time point for every subject has been chosen. The numbers denote the time point that has been used in the validation set.

Combination	Timepoint in validation
First	last
Second	3
Third	2
Fourth	1

Table A5. Challenge results for different filter sets and loss functions. This table illustrates the performance metrics of the main architecture trained with different filter sets and loss functions, evaluated by the challenge submission [2].

Filter Range	Loss Function	DSC	PPV	TPR	LFPR	LTPR	Submission Score
**64 - 1024**	DICE	*0.64*	0.86	*0.56*	0.21	**0.57**	*92.637*
**32 - 512**	DICE	**0.66**	0.84	**0.58**	0.22	*0.55*	92.461
**32 - 512**	DICE + BCE	*0.64*	0.85	*0.56*	*0.20*	*0.55*	**92.661**
**32 - 512**	L2	0.60	**0.90**	0.49	**0.14**	0.46	92.41
**32 - 512**	FOCAL	0.63	*0.87*	0.54	0.24	0.53	92.136

Table A6. Rater-to-consensus-mask metrics for the MSSEG data. This table illustrates the resulting metrics from the comparison of every label mask from the seven different raters with the consensus mask that was created out of all those seven label masks from the MSSEG Challenge. Metrics were calculated with the evaluation software of the ISBI Challenge [4].

	Consensus mask						
**Rater**	**DSC**	**Jaccard**	**PPV**	**TPR**	**LFPR**	**LTPR**	**VD**
**1**	0.7689	0.6307	0.7582	0.8015	0.1701	0.8881	0.2169
**2**	0.7078	0.5704	0.6276	0.8253	0.2377	0.8476	0.3364
**3**	0.7011	0.5503	0.7291	0.7038	0.5170	0.7958	2594
**4**	0.6880	0.5510	0.5997	0.8341	0.2342	0.8860	0.4966
**5**	0.7760	0.6389	0.6895	0.9034	0.1809	0.9371	0.3592
**6**	0.6964	0.5549	0.7579	0.6767	0.3450	0.7123	0.2458
**7**	0.7020	0.5530	0.8277	0.6327	0.4719	0.7480	0.2811

Table A7. Results of transfer learning after every fifth epoch. This table compares the results of two architectures after the transfer learning on the MSSEG dataset. The transfer learning was done according to table A2. Both networks were initially trained on the ISBI set [2]. The numbers are averaged over all subjects. The best results per network architecture are written in bold.

Networks trained on Dice Loss		Consensus mask					
**Filter range**	**Epoch**	**DSC**	**PPV**	**TPR**	**LFPR**	**LTPR**	**VD**
**32-512**	0	0.68 [0.64, 0.71]	0.75 [0.71, 0.80]	0.64 [0.60, 0.68]	0.60 [0.55, 0.64]	0.64 [0.59, 0.68]	**0.29** [0.25, 0.32]
**32-512**	5	0.71 [0.68, 0.75]	**0.76** [0.72, 0.80]	0.71 [0.67, 0.74]	0.53 [0.48, 0.57]	0.70 [0.65, 0.74]	0.30 [0.21, 0.38]
**32-512**	10	**0.72** [0.68, 0.75]	0.75 [0.71, 0.79]	**0.72** [0.68, 0.75]	0.53 [0.49, 0.58]	0.71 [0.67, 0.76]	0.30 [0.20, 0.41]
**32-512**	15	0.72 [0.68, 0.75]	0.75 [0.71, 0.80]	0.72 [0.68, 0.75]	0.53 [0.49, 0.58]	**0.71** [0.67, 0.76]	0.31 [0.20, 0.41]
**32-512**	20	0.72 [0.68, 0.75]	0.76 [0.72, 0.80]	0.71 [0.67, 0.74]	**0.52** [0.48, 0.56]	0.71 [0.66, 0.75]	0.30 [0.21, 0.39]
64 - 1024	0	0.68 [0.65, 0.71]	**0.77** [0.73, 0.81]	0.63 [0.59, 0.67]	0.63 [0.58, 0.68]	0.64 [0.60, 0.68]	0.29 [0.25, 0.32]
**64 - 1024**	5	0.68 [0.63, 0.73]	0.74 [0.68, 0.80]	0.68 [0.65, 0.72]	0.52 [0.47, 0.57]	0.69 [0.64, 0.73]	0.61 [0.30, 0.91]
**64 - 1024**	10	**0.69** [0.65, 0.73]	0.75 [0.70, 0.81]	**0.69** [0.65, 0.72]	**0.51** [0.45, 0.56]	0.70 [0.66, 0.74]	0.43 [0.24, 0.61]
**64 - 1024**	15	0.69 [0.64, 0.73]	0.75 [0.69, 0.80]	0.68 [0.64, 0.72]	0.51 [0.46, 0.56]	0.70 [0.66, 0.74]	0.49 [0.27, 0.70]
**64 - 1024**	20	0.68 [0.64, 0.73]	0.75 [0.69, 0.80]	0.69 [0.65, 0.72]	0.51 [0.45, 0.56]	**0.70** [0.66, 0.75]	0.55 [0.28, 0.81]

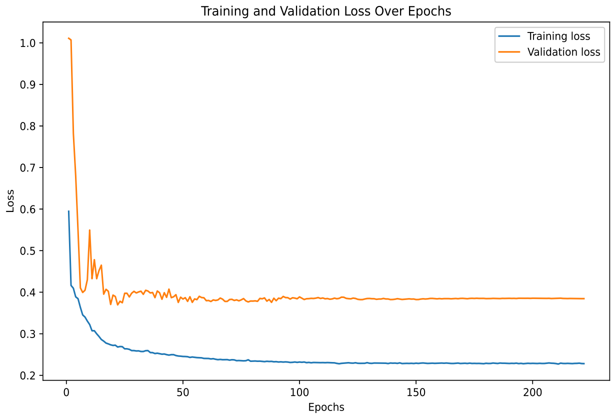

Figure A1. Loss curves from training This figure illustrates the loss curves from one combination of the LOOCV during training.

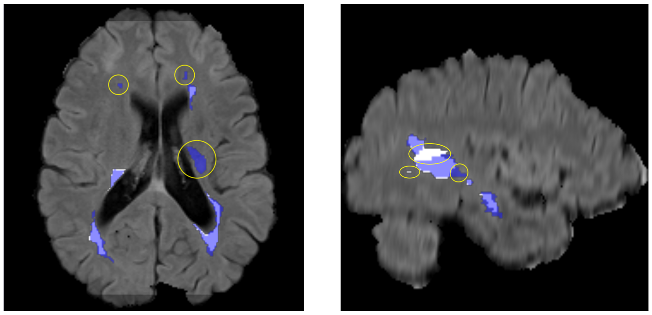

Figure A2. FLAIR Scan of the ISBI dataset with annotations from both raters. This figure illustrates an axial (left) and sagittal (right) slice from a FLAIR scan of the first time point from Subject 1 in the ISBI training set with the annotated delineations from the two different raters.[2] The first rater's annotations are white, the second one's are a dark blue and where they overlap they mix to a light blue. Labeled areas with no overlap between the two raters at all are marked with yellow circles/ellipses.

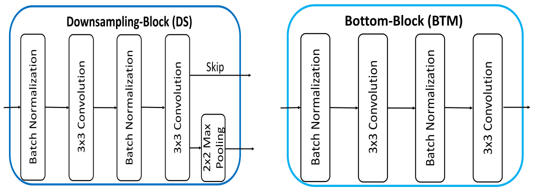

Figure A3. **Down-sampling and bottom block of the proposed architecture.** This figure illustrates the proposed down-sampling- (*left*) and bottom-blocks (*right*). The output of the convolutions are also sent via skip connections to the MMFF-blocks (see figure A4 (*left*)). The MSFU-blocks (see figure A4 (*right*)) take the output from one MMFF-block, as well as from another MSFU-block (or the bottom-block) as an input. The last MSFU-block is followed by a 1×1 convolution layer with a sigmoidal activation function.

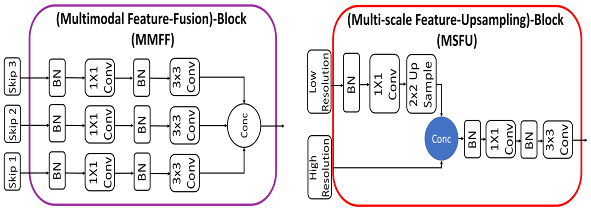

Figure A4. **MMFF- and MSFU-blocks.** This figure illustrates the proposed MMFF- *(left)* and MSFU-blocks *(right)*.

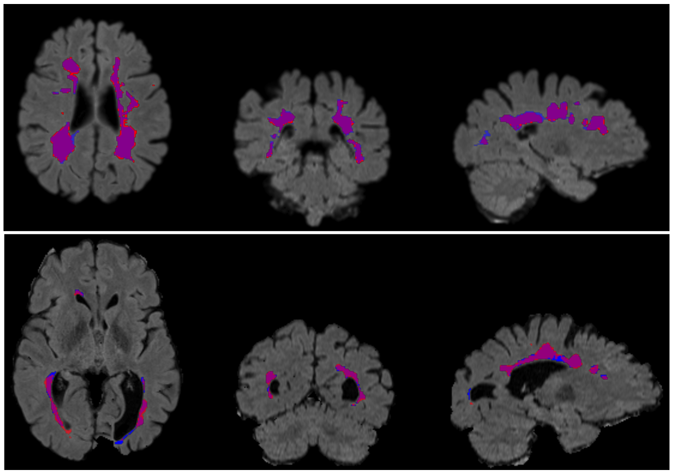

Figure A5. **Predictions on a Subject of the ISBI dataset with a high lesion load.** This figure illustrates the predictions on the fourth time point of subject 2 from the ISBI training set [5] with *top*: a high lesion load and *bottom*: a low lesion load. From left to right, there is the axial, coronal and the sagittal view. Blue are the annotations from rater 1, red are the predictions from the network and purple shows, where they overlap.

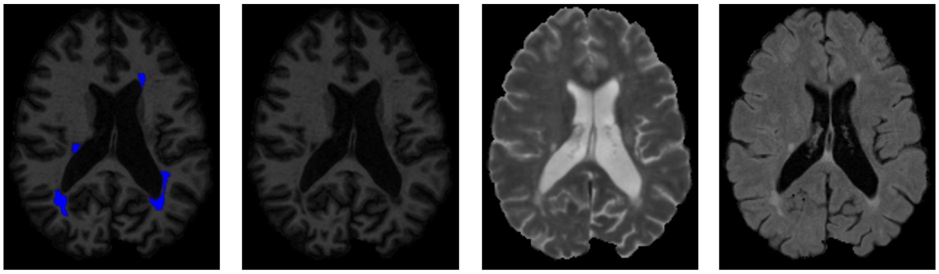

Figure A6. **Labelled/unlabelled axial slice of different modalities.** This figure shows an axial Slice of the different modalities from the fourth time point of the first Subject in the ISBI Train dataset. From left to right, there is a T1w Scan with the annotated lesions (blue), the same T1w Slice, where some of the lesions appear as black-holes. In the *third* image, one can see a T2w image of the same slice, where the lesions appear as hyper-intensities and the *fourth* image shows a FLAIR scan, where the lesions are also hyper-intensities.

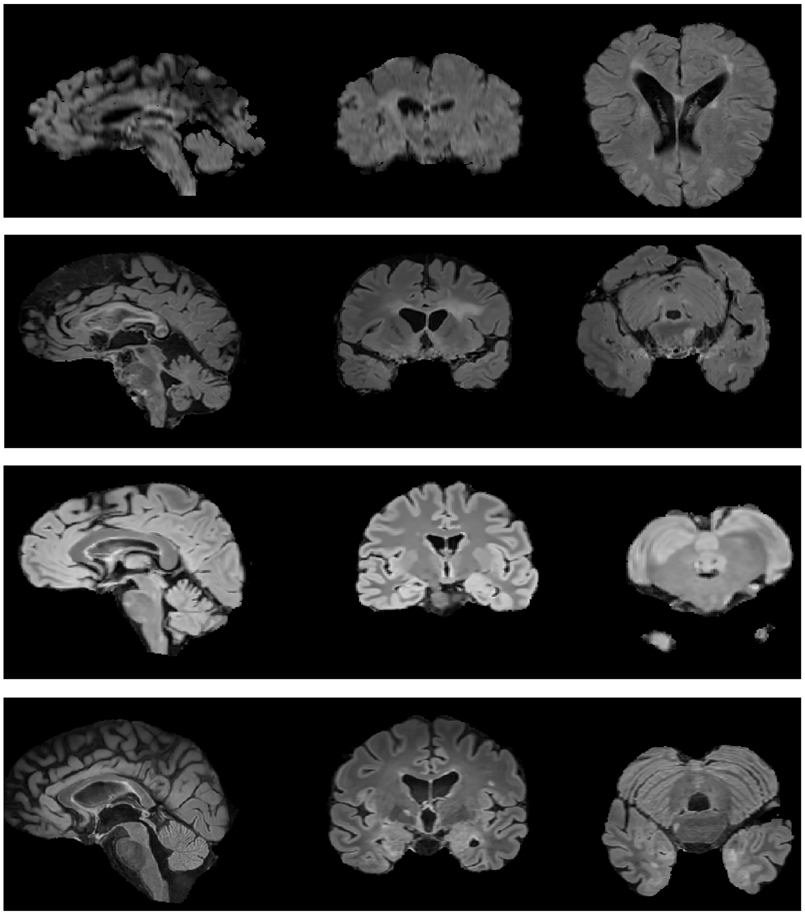

Figure A7. **Illustration of scanner variability for FLAIR scans.** This figure illustrates the scanner variability, based on four FLAIR scans that were acquired on four different scanners. From top to bottom, the *first* is a 2D FLAIR MRI of subject 1, first time point from the ISBI dataset, acquired on a 3T Philips MRI scanner. The *second* is a 3D FLAIR MRI of subject 01016SACH from the MSSEG dataset, acquired on a Siemens 3T Verio MRI scanner. In the *third* image, there is a 3D FLAIR MRI of subject 07001MOEL from the MSSEG dataset, acquired on a Siemens 1.5T Aera scanner. The *fourth* image shows a 3D FLAIR MRI of subject 08002CHJE from the MSSEG dataset, acquired on a Philips 3T Ingenia scanner.

References

1. Kirch, W. (ed.). *Pearson's Correlation Coefficient*, 1090–1091 (Springer Netherlands, Dordrecht, 2008).

2. Carass, A. *et al.* Longitudinal multiple sclerosis lesion segmentation: Resource and challenge. *NeuroImage* **148**, 77–102, DOI: https://doi.org/10.1016/j.neuroimage.2016.12.064 (2017).

3. Commowick, O. *et al.* Objective evaluation of multiple sclerosis lesion segmentation using a data management and processing infrastructure. *Sci. Reports* **8**, 13650, DOI: 10.1038/s41598-018-31911-7 (2018).

4. ISBI. Challenge metrics. http://iacl.ece.jhu.edu/index.php?title=MSChallenge/evaluation.

5. ISBI. Dataset description. https://smart-stats-tools.org/sites/default/files/lesion_challenge/Training_data_description.pdf (2015).

List of Figures in Appendix

A1 Loss curves from training 4
A2 FLAIR Scan of the ISBI dataset with annotations from both raters 4
A3 Down-sampling and bottom block of the proposed architecture 5
A4 MMFF- and MSFU-blocks 5
A5 Predictions on a Subject of the ISBI dataset with a high lesion load 5
A6 Labelled/unlabelled axial slice of different modalities 6
A7 Illustration of scanner variability for FLAIR scans 6

List of Tables in Appendix

A1 Evaluation protocol with ISBI 2015 challenge training data 2
A2 Transfer learning protocol 2
A3 Comparison of different filter sets in the main architecture 3
A4 Choice of datasets for training the networks, used for challenge submission 3
A5 Challenge results for different filter sets and loss functions 3
A6 Rater-to-consensus-mask metrics for the MSSEG data 3
A7 Results of transfer learning after every fifth epoch 4
